# SNPs Associated With Testosterone Levels Influence Human Facial Morphology

**DOI:** 10.3389/fgene.2018.00497

**Published:** 2018-10-23

**Authors:** Jasmien Roosenboom, Karlijne Indencleef, Myoung Keun Lee, Hanne Hoskens, Julie D. White, Dongjing Liu, Jacqueline T. Hecht, George L. Wehby, Lina M. Moreno, Carolyn Hodges-Simeon, Eleanor Feingold, Mary L. Marazita, Stephen Richmond, Mark D. Shriver, Peter Claes, John R. Shaffer, Seth M. Weinberg

**Affiliations:** ^1^Department of Oral Biology, Center for Craniofacial and Dental Genetics, University of Pittsburgh, Pittsburgh, PA, United States; ^2^ESAT-PSI, Department of Electrical Engineering, Medical Imaging Research Center, KU Leuven, Leuven, Belgium; ^3^Department of Anthropology, Penn State University, University Park, PA, United States; ^4^Department of Human Genetics, University of Pittsburgh, Pittsburgh, PA, United States; ^5^Department of Pediatrics, University of Texas McGovern Medical Center, Houston, TX, United States; ^6^Department of Health Management and Policy, University of Iowa, Iowa City, IA, United States; ^7^Department of Orthodontics, University of Iowa, Iowa City, IA, United States; ^8^Department of Anthropology, Boston University, Boston, MA, United States; ^9^Applied Clinical Research and Public Health, School of Dentistry, Cardiff University, College of Biomedical and Life Sciences, Cardiff, United Kingdom

**Keywords:** testosterone, facial morphology, candidate SNP, SHBG, facial ratio, ALSPAC

## Abstract

Many factors influence human facial morphology, including genetics, age, nutrition, biomechanical forces, and endocrine factors. Moreover, facial features clearly differ between males and females, and these differences are driven primarily by the influence of sex hormones during growth and development. Specific genetic variants are known to influence circulating sex hormone levels in humans, which we hypothesize, in turn, affect facial features. In this study, we investigated the effects of testosterone-related genetic variants on facial morphology. We tested 32 genetic variants across 22 candidate genes related to levels of testosterone, sex hormone-binding globulin (SHGB) and dehydroepiandrosterone sulfate (DHEAS) in three cohorts of healthy individuals for which 3D facial surface images were available (Pittsburgh 3DFN, Penn State and ALSPAC cohorts; total *n* = 7418). Facial shape was described using a recently developed extension of the dense-surface correspondence approach, in which the 3D facial surface was partitioned into a set of 63 hierarchically organized modules. Each variant was tested against each of the facial surface modules in a multivariate genetic association-testing framework and meta-analyzed. Additionally, the association between these candidate SNPs and five facial ratios was investigated in the Pittsburgh 3DFN cohort. Two significant associations involving intronic variants of *SHBG* were found: both rs12150660 (*p* = 1.07E-07) and rs1799941 (*p* = 6.15E-06) showed an effect on mandible shape. Rs8023580 (an intronic variant of *NR2F2-AS1*) showed an association with the total and upper facial width to height ratios (*p* = 9.61E-04 and *p* = 7.35E-04, respectively). These results indicate that testosterone-related genetic variants affect normal-range facial morphology, and in particular, facial features known to exhibit strong sexual dimorphism in humans.

## Introduction

Differences in facial morphology between males and females are well documented, with major shape differences apparent in the jaw, lips, eyes, nose and cheek regions (Toma et al., 2008; Klotz et al., 2010; Claes et al., 2012b; Koudelová et al., 2015). Although facial sexual dimorphism has now been described in children (Kesterke et al., 2016; Matthews et al., 2016, 2018), differences become much more pronounced after the onset of puberty. This accelerated dimorphism post-puberty is the result of changes in circulating hormone levels, which regulate the development and differentiation of male and female primary and secondary sex characteristics, such as voice, body shape and facial morphology (Hines, 2011). In fact, testosterone levels are 20–30 times higher in males than in females during this developmental stage (Duncan et al., 2009).

Several studies showed a relationship between testosterone levels (either measured directly or through proxy) and facial morphology, using several different study set-ups and approaches. For example, the second to fourth digit ratio (2D:4D), which is a marker of prenatal androgen exposure, corresponds to measures of facial masculinity and sexually dimorphic facial features (Schaefer et al., 2005; Meindl et al., 2012). Similarly, Whitehouse et al. (2015) investigated the effect of prenatal testosterone exposure (measured in umbilical cord blood) on postnatal facial morphology, and showed that higher cord testosterone levels were associated with masculinized facial features. Hodges-Simeon et al. (2016) showed that facial width/lower facial height decreases, cheekbone prominence decreases, and lower face height/full face height increases under the influence of pubertal testosterone. In a study by Marečková et al. (2011), facial appearance was rated to be more masculine in males with a higher level of bioavailable testosterone. Other evidence for the influence of testosterone on facial morphology comes from the craniofacial differences in boys with delayed puberty before and after testosterone treatment. Verdonck et al. (1999) described an accelerated craniofacial growth after testosterone treatment, especially in total mandibular length, ramus length, and upper and anterior facial height.

The amount of circulating testosterone present in the body is the result of many factors, including genetics. Genome-wide association studies (GWAS) have identified multiple variants that influence testosterone regulation in healthy adults (Ohlsson et al., 2011; Zhai et al., 2011; Coviello et al., 2012; Jin et al., 2012; Prescott et al., 2012; Chen et al., 2013; Ruth et al., 2016), including variants in SHBG, the gene encoding sex-hormone binding globulin. We hypothesize that these variants may also be associated with aspects of facial morphology, particularly those features that demonstrate strong evidence of sexual dimorphism in humans. In this study, we investigated the effect of previously identified genetic variants involved in testosterone regulation on normal-range human facial shape in 7418 European individuals belonging to three cohorts with available 3D facial and genomic data. Additionally, the association between these candidate SNPs and five facial ratios was investigated in a subset of individuals; these ratios have been shown in the literature to correlate with testosterone levels or testosterone-related traits (e.g., aggressive behavior). As a consequence, this study has the potential to shed new light on the biological basis of human facial sexual dimorphism and, more generally, normal-range facial shape variation.

## Materials and Methods

### Study Sample

The study sample consists of 7418 individuals belonging to three separate cohorts: the Pittsburgh 3D Facial Norms (3DFN) cohort (Weinberg et al., 2016), the Penn State cohort and the Avon Longitudinal Study of Parents and their Children (ALSPAC) cohort (Boyd et al., 2013; Fraser et al., 2013). The Pittsburgh 3DFN cohort consists of 2297 participants (age 3–40 years) of self-reported Western-European descent without a history of facial defects or craniofacial surgery. These individuals were recruited at four United States sites: Pittsburgh, PA; Seattle, WA; Houston, TX and Iowa City, IA. The Penn State cohort consists of 1555 subjects (age 18–83 years) of European descent without a history of facial defects or craniofacial surgery, recruited from a number of United States and international sites: State College, PA; New York, NY; Urbana-Champaign, IL; Twinsburg, OH; Dublin, Ireland; Rome, Italy; Warsaw, Poland and Porto, Portugal. 3566 subjects of the Avon Longitudinal Study of Parents and their Children (ALSPAC) cohort (age 14–17 years) were included in this study These subjects are of Western–European descent without a history of facial defects or craniofacial surgery, recruited in Avon, England. ALSPAC recruited 14541 pregnant women resident in Avon, United Kingdom with expected dates of delivery 1st April 1991 to 31st December 1992. 14541 is the initial number of pregnancies for which the mother enrolled in the ALSPAC study and had either returned at least one questionnaire or attended a “children in focus” clinic by 19/07/1997. Of these initial pregnancies, there was a total of 14676 fetuses, resulting in 14062 live births and 13988 children who were alive at 1 year of age. Please note that the study website contains details of all the data that is available through a fully searchable data dictionary^1^. Genome-wide data was available for 8,952 subjects of the B2261 study which is titled “Exploring distinctive facial features and their association with known candidate genes.”

### Defining Facial Phenotypes

3D facial surface images were acquired using multiple stereophotogrammetry systems: the VECTRA H1 camera (Canfield Scientific, Parsippany, NJ, United States) and the 3dMDface system (3dMD, Atlanta, GA, United States) were used for the Pittsburgh 3DFN and Penn State cohorts, and the Konica Minolta Vivid 900 laser scanner (Konica Minolta Sensing Europe, Milton Keynes, United Kingdom) was used for the ALSPAC cohort. The process of registration, quality control and segmentation is described in detail in Claes et al. (2018). Briefly, the 3D images were imported in Matlab™ 2016b for a spatial-dense registration of the images (Claes et al., 2012a), in which a symmetrical anthropometric mask was mapped onto the images, leading to a homologous spatial-dense configuration of quasi-landmarks. Subsequently, a two-step image quality control was performed: first, outlier faces were identified by calculating the z-scores from the Mahalanobis distance between the mean face and each subject, and subjects with z-scores higher than two were manually checked; second, a score was calculated that reflects the missing data in the images (due to mesh artifacts or holes) and images with a high score were also manually checked and removed from the dataset when necessary. The spatial-dense configurations then underwent a generalized Procrustes superimposition to eliminate differences in orientation, position and scale (Rohlf and Bookstein, 2003). Analyses were done using symmetrized images, created by averaging the original and reflected images (created by changing the sign of the x-coordinate of the original mapped images).

**FIGURE 1:**
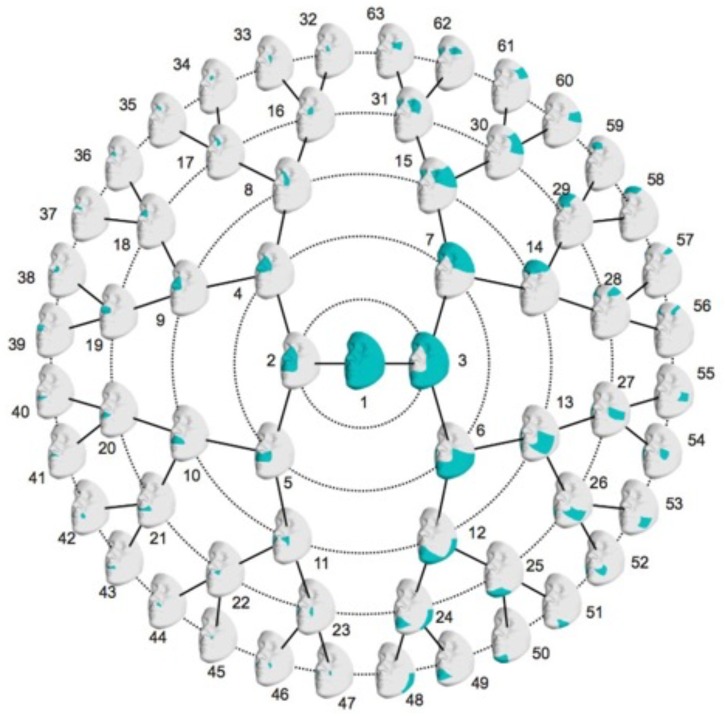
Rosette diagram showing the hierarchical global-to-local facial segmentation scheme. A total of 63 facial modules are represented. Modules are indicated in turquoise.

The images were adjusted for the effects of age, age^2^, sex, weight, height, facial size and the first four principal components (PCs) of genetic ancestry using partial least-squares regression. The images were adjusted in each dataset separately. After the adjustment, segmentation was performed on all three datasets combined to develop multivariate facial phenotypes representing specific facial modules. Briefly, facial modules were defined by grouping vertices that are strongly correlated and connected using hierarchical spectral clustering. The strength of co-variation between the modules was defined using Escoufier’s RV coefficient (Robert and Escoufier, 1976), which is a scalar measure of the strength of association between two groups of variables and is used in morphometric studies on biological shapes (Klingenberg, 2009). The RV coefficient allowed us to build a structural similarity matrix that defined the hierarchical construction of 63 facial modules, which is represented in Figure 1. Subsequently, for each of the 63 modules, quasi-landmarks were aligned via generalized Procrustes superimposition, followed by principal component analysis (PCA) to reduce the dimensionality of the data, and finally parallel analysis to determine the number of PCs needed to adequately capture the shape variation (Hayton et al., 2004). These linear combinations of PCs formed the multivariate shape phenotypes to be used in genetic association testing (described below).

### Genotyping and Quality Control

Genotyping and quality control of the Pittsburgh 3DFN and Penn State cohorts were previously described by Claes et al. (2018). Briefly, for the Pittsburgh 3DFN cohort, DNA was extracted from saliva samples and genotyped along with 72 HapMap control samples on the Illumina HumanOmniExpress + Exome v1.2 array (Illumina, San Diego, CA, United States) plus custom content by the Center for Inherited Disease Research (CIDR). Samples were interrogated for genetic sex, chromosomal aberrations, relatedness, genotype call rate, and batch effects. SNPs were interrogated for call rate, discordance among 70 duplicate samples, Mendelian errors among HapMap controls (parent-offspring trios), deviations from Hardy-Weinberg equilibrium, and sex differences in allele frequencies and heterozygosity. To assess population structure, we performed a PCA within each cohort using subsets of uncorrelated SNPs. Based on the scatterplots of the PCs and scree plots of the eigenvalues, we determined that population structure was captured in four PCs of ancestry for the 3DFN cohort. Imputation of unobserved variants was performed using haplotypes from the 1000 Genomes Project Phase 3 as the reference. Imputation was performed using IMPUTE2 (Howie et al., 2009). We used an info score of >0.5 at the SNP level and a genotype probability of >0.9 at the SNP-per-participant level as filters for imputed SNPs. Masked variant analysis, in which genotyped SNPs were imputed in order to assess imputation quality, indicated high accuracy of imputation.

Participants in the Penn State cohort were genotyped using the Illumina Human Hp200c1 BeadChip; and the 23andMe v3 and v4 arrays. Samples were interrogated for genetic sex, chromosomal aberrations, relatedness, genotype call rate, and batch effects. SNPs were evaluated for call rate, discordant genotype calls between duplicate samples, Mendelian errors in HapMap control parent–offspring trios, deviation from Hardy–Weinberg genotype proportions and sex differences in allele frequency and heterozygosity. Imputation was performed as described for the 3DFN cohort. An ADMIXTURE analysis was done with the 1000 Genomes Phase 1 dataset as the reference, to select subjects with European ancestry (Alexander et al., 2009).

Participants from the ALSPAC cohort were genotyped using the Illumina HumanHap550 quad genome-wide SNP genotyping platform, by Sample Logistics and Genotyping Facilities at the Wellcome Trust Sanger Institute (Cambridge, United Kingdom) and the Laboratory Corporation of America (Burlington, NC, United States), supported by 23andMe. Haplotypes were estimated using SHAPEIT2 (Delaneau et al., 2013) and imputed to the 1000 genomes reference panel (Phase 1, Version 3) (Abecasis et al., 2012) using IMPUTE2 (Howie et al., 2009, 2011).

The following procedure was used to identify and exclude biological relatives in the ALSPAC cohort. First, SNPs with a minor allele frequency (MAF) less than 5% or more than 5% genotype data missing, were filtered out. Second, SNPs were pruned for linkage disequilibrium (LD) with r^2^ set at 0.2 in a pairwise manner, with a moving window size of 50 variants shifting five variants each step. Finally, related individuals were identified and removed when the proportion of identity by descent (IBD) was higher than 0.125. To control for population structure, self-identified non-European individuals were removed, and then genetic outliers (*n* = 15) were removed based on z-scores calculated in the first 10 PCs. Z-scores higher than six indicated outliers, who were subsequently removed after which PCA was computed again. These calculations were done using PLINK 1.9 (Purcell et al., 2007). After these steps, ancestry axes were determined with PCA and used as described above.

### Candidate Genes

SNPs reported to influence testosterone levels in the human body were identified from the literature. Since free circulating testosterone levels are variable and not easy to measure, proxy-measurements have been used to estimate the levels of free testosterone in the human body. Dehydroepiandrosterone (DHEAS) is the most prevalent circulating steroid in humans (Vandenput and Ohlsson, 2014) and is a precursor for testosterone. Furthermore, SHBG binds to free circulating testosterone and estradiol and is therefore a good proxy for testosterone levels in the body. Therefore, genetic studies of testosterone regulation have focused on free circulating testosterone, DHEAS, and/or SHBG. Forty-three testosterone-related SNPs were selected based on genetic association results from seven publications (Ohlsson et al., 2011; Zhai et al., 2011; Coviello et al., 2012; Jin et al., 2012; Prescott et al., 2012; Chen et al., 2013; Ruth et al., 2016; Supplementary Table S1). 32 of these SNPs were present in all three cohorts and were tested for association with the 63 facial modules. Three SNPs were not tested because of MAF < 5% in one or more cohorts and the other SNPs were excluded due to low imputation quality in one or more cohorts.

### Statistical Testing and Meta-Analysis

Canonical correlation analysis (CCA) was used to test the effect of 32 SNPs on facial shape using the multivariate phenotypes derived as described above. CCA is a multivariate testing framework which extracts the linear combination of PCs from a facial module that has maximal correlation with the SNP being tested. The correlation was tested for significance by a Roa’s *F*-test approximation (right tail, one-sided test), after removing the effects of age, age^2^, sex, weight, height, facial size and the first four dimensions of ancestry using PLSR. Both the independent (SNP) as the dependent (facial shape) variables were corrected for these covariates.

Since the direction of the effect through the face shape space determined by the CCA can and will differ across the three cohorts, we use a two-step procedure where CCA is performed in one cohort, after which the two other cohorts were projected onto the direction of the effect found in CCA. Thus, three meta-analyses were performed in a round-robin fashion with each of the three cohorts serving as the discovery set for the CCA and the other two serving as replication sets. Association tests were performed in the replication sets by projecting the PCs onto the loadings calculated in the discovery CCA, which returned a specific genetic effect score. As described in Claes et al. (2018), the phenotypic trait discovered in CCA, can be explicitly measured in the replication datasets. The genetic effect score was subsequently used in a standard linear regression with the SNPs as independent variables. This function employs a t-statistic and a positive-sided *p*-value was obtained with the Student’s T cumulative distribution function (Devroye, 1986). The *p*-values were combined according to Stouffer’s method (1949). The facial segments are overlapping and hierarchically constructed (thus not completely independent). Therefore we determined 37 independent segments by the Li and Ji method (Li and Ji, 2005). Thirty-two candidate SNPs were tested, of which 30 were independent. An additional correction factor was added due to the three meta-analyses performed. Therefore, the Bonferroni-corrected significance *p*-value cut-off was *p* < 1.50E-05 (i.e., 0.05/(37×30×3)).

Analyses were repeated for males (*n* = 4055) and females (*n* = 2892) separately, and in a strictly post-pubertal cohort (age > 15 years; *n* = 6947).

### Investigating Alternative Phenotypes: Facial Ratios

Several standard facial ratios have been previously associated with either testosterone levels or testosterone-related phenotypes (e.g., aggression, perceived masculinity) (Carré et al., 2010; Haselhuhn and Wong, 2012; Geniole et al., 2015; Hodges-Simeon et al., 2016). We therefore looked at the relationship between our candidate SNPs and five facial ratio measurements in the Pittsburgh 3DFN cohort (*n* = 2297), including standard facial width-to-height ratios (Supplementary Figure S1). These ratios were calculated based on linear distance measurements collected directly on the 3D facial images or through direct anthropometry (see Weinberg et al., 2016 for details).

A total of 42 of the 43 literature-identified SNPs associated to testosterone levels were available for testing in the Pittsburgh 3DFN cohort (genotyping and quality control of this cohort is described above). All five ratios were adjusted by sex, age and body size before genetic analysis, then linear models were used to test genetic association between each phenotype and each SNP adjusting for the first four PCs of ancestry using PLINK. The Bonferroni-corrected significance *p*-value cut-off was *p* < 0.00119 (i.e., 0.05/42). For completeness, associations in subgroups of the total study population were also investigated: males only (*n* = 856), females only (*n* = 1323), and post-pubertal (>14 years) participants (*n* = 1750).

## Results

### Association of Testosterone-Associated SNPs With Facial Shape Modules

**FIGURE 2:**
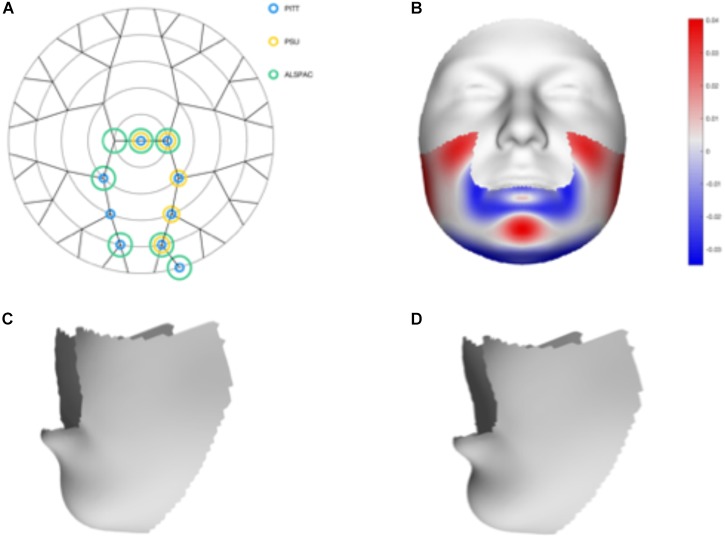
Meta-analysis results for rs12150660. **(A)** Representation of the significant modules of the meta-analysis based on the different discovery datasets. **(B)** Heat map displacement plot of the effect of rs12150660 on facial morphology, with red representing an outward displacement and blue representing an inward displacement. **(C)** Surface warp showing the effect (exaggerated) of the major allele (G) on lower facial shape. **(D)** Surface warp showing the effect (exaggerated) of the minor allele (T) on lower facial shape.

**FIGURE 3:**
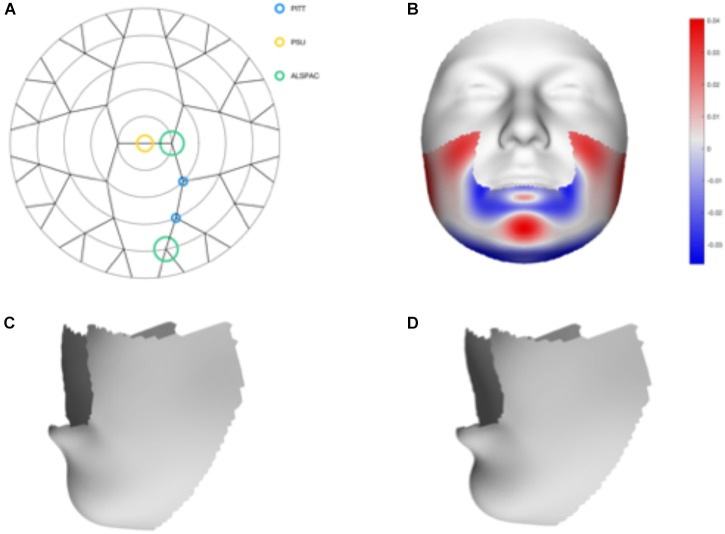
Meta-analysis results of rs1799941. **(A)** Representation of the significant modules of the meta-analysis based on the different discovery datasets. **(B)** Heat map displacement plot of the effect of rs1799941 on facial morphology, with red representing an outward displacement and blue representing an inward displacement. **(C)** Surface warp showing the effect (exaggerated) of the major allele (G) on lower facial shape. **(D)** Surface warp showing the effect (exaggerated) of the minor allele (A) on lower facial shape.

We tested 32 SNPs for genetic association with 63 multivariate facial modules. Two of the 32 tested SNPs showed significant associations with modules in the mandible region in at least one of the three meta-analyses: rs12150660 (*p* = 1.07E-07) and rs1799941 (*p* = 6.15E-06) (Table 1 and Figures 2, 3). Both of these SNPs are located in introns of the *SHBG* gene, and have been shown to affect circulating SHBG as previously described in literature (Ohlsson et al., 2011; Jin et al., 2012). SHBG is a binding globulin secreted by the liver and its major function is to bind and transport circulating sex hormones with a high affinity; transporting them into the circulation and regulating their action by controlling their bioavailability (Coviello et al., 2012; Vandenput and Ohlsson, 2014). In the circulation, 50–60% of the free testosterone is bound to SHBG, while only 1–2% is free-circulating (Jin et al., 2012). Low levels of circulating SHBG are associated with decreased glucose control and are predictors for type 2 diabetes (Kuijper et al., 2007). Subjects with the minor allele of rs12150660 were more likely to show lower testosterone levels (Ohlsson et al., 2011), while subjects with the minor allele of rs1977741 showed higher testosterone levels (Jin et al., 2012). The minor alleles of both SNPs were associated with a broader and more protruding mandible – a characteristic feature associated with male faces. While the effects of rs1799941 were localized to the mandibular region, rs12150660 showed additional effects in modules containing the philtrum and was associated specifically with midfacial retrusion or a more protruded chin (Figures 2, 3). Since both identified SNPs are in LD (*r*^2^ = 0.89), it is possible that only one of them is actually functional, or both of them may be in strong LD with yet another untested causal variant (Ohlsson et al., 2011). This could mean that, although being in high LD, both SNPs might still have a separate effect on SHBG and testosterone levels in the body, reflected in the face. The same phenotypic effect of these SNPs was observed in the post-pubertal subset (Supplementary Figure S2). No significant associations were observed in the male- and female-only subsets. Meta-analysis results for all 32 SNPs are displayed in Supplementary Table S2.

**Table 1:** Discovery and meta-analysis results of rs12150660 and rs1799941 for the 3D facial modules.

SNP	Location (hg19)	Candidate gene	Allele	MAF	Module	CCA		Meta-analysis
						CC	*P*-value	*P*-value
rs12150660	17:7618597	SHBG	G > T	0.0925	6	0.163	2.02E-02	1.07E-07
						0.178	1.67E-01	7.08E-06
						0.154	7.65E-05	5.03E-05
rs1799941	17:7630105	SHBG	G > A	0.10	12	0.145	1.70E-02	6.15E-06
						0.129	6.87E-01	9.80E-04
						0.137	3.41E-04	2.08E-03

*For the allele column, major allele > minor allele. MAF = minor allele frequency (based on 1000 genomes phase 3 for Europeans). Genome build hg19 is used as standard reference genome sequence to indicate the genomic location of the variants. The module number refers to Figure 1 and shows the facial segment with the lowest p-value for the SNP. CCA shows the canonical correlation (CC) analysis results for the module listed. Results are ordered by row according to which cohort served as discovery in the meta-analysis: Pittsburgh, Penn State, ALSPAC. The meta-analysis p-values are ordered in the same manner. The first p-value (under CCA) represents the statistical significance observed for the cohort separately. The second p-value (under meta-analysis) represents with combined statistical significance observed with each cohort serving in the discovery position.*

### Testing Facial Ratios

Based on the findings of Hodges-Simeon and others, who have showed an association between pubertal testosterone levels and ratios of facial width-to-height (Lefevre et al., 2013; Hodges-Simeon et al., 2016), we tested five facial ratios in our candidate gene approach. The genetic associations between the candidate SNPs and the ratios (Supplementary Table S3) were investigated in the entire 3DFN sample, in males/females separately, and in a post-pubertal subpopulation. One of the 42 testosterone SNPs we tested (rs8023580) showed an association with two related facial ratios in the male-only subset: total facial width-to-height (*p* = 9.61E-04) and upper facial width-to-height (FWH1; *p* = 7.35E-04). rs8023580 is an intronic SNP in *NR2F2-AS1*, a non-coding RNA showing high expression in the ovary. The complete results are shown in Supplementary Table S3. The nearby *NR2F2* gene has been implicated in testicular Leydig function, essential for male testosterone production (Hu et al., 2013). Furthermore, several animal studies suggest an influence of *NR2F2* on craniofacial morphology. *NR2F2* is expressed in the mandibular component of the first branchial arch in mice. In zebrafish, it has been reported that *NR2F* genes are required for upper jaw formation (Barske et al., 2018). Furthermore, loss of these genes transforms the maxillary component of the upper jaw into lower-jaw-like structures. Dieterich et al. (2015) suggested that the expression of *NR2F2* might be regulated by *NR2F2-AS1* via *MAFB*, which is a gene known to be involved in craniofacial development (Dieterich et al., 2015). In a recent GWAS of human facial morphology, variants near *MAFB* were shown to be associated with overall width of the cranial base (Shaffer et al., 2016). This measure is a reasonable proxy for upper facial width, which is a key determinant of the facial width-to-height ratios associated with *NR2F2-AS1* variants here.

## Discussion

The impact of sex hormones on human craniofacial morphology is well documented and is most apparent in the post-pubertal dimorphism we see between male and female faces (Kesterke et al., 2016; Matthews et al., 2018). The most prominent sex differences in human facial morphology tend to involve the mandible, zygomatic region (cheeks), lips, forehead, and nose (Toma et al., 2008; Koudelová et al., 2015; Kesterke et al., 2016; Matthews et al., 2018). Prior studies have connected these aspects of facial morphology to both testosterone levels directly or to other physical and behavioral markers of testosterone activity (Verdonck et al., 1999; Whitehouse et al., 2015; Hodges-Simeon et al., 2016). If this causal model is correct, then any factor affecting testosterone levels should also impact facial morphology along with a host of other sexually dimorphic traits. We now understand that the level of free testosterone in individuals is at least partly under genetic control (Ohlsson et al., 2011; Zhai et al., 2011; Coviello et al., 2012; Jin et al., 2012; Prescott et al., 2012; Chen et al., 2013; Ruth et al., 2016). In the present study, we performed an *in silico* candidate gene study to investigate the effects of common genetic variants known to impact testosterone levels on normal-range facial morphology. The SNPs we chose were previously reported in several large-scale GWAS to influence free testosterone levels in adults. We observed associations with three of these SNPs in two genes: *SHBG* and *NR2F2-AS1*. These SNPs were shown to impact specific regions of the face that show strong evidence of sex differences – the mandible, lips, and upper face. The lower third of the face (mandibular region), in particular, has been shown to correlate strongly with traits related to prenatal testosterone exposure, such as second-to-fourth digit ratio. In prior research, individuals with lower digit ratios (indicating greater prenatal testosterone exposure) showed broader, more robust, and more protrusive mandibles (Meindl et al., 2012; Weinberg et al., 2015). In contrast, nasal morphology, which often shows striking differences post-puberty between males and females (Kesterke et al., 2016), was not associated with these or any other SNPs we tested.

The mechanism of effect of the implicated variants on facial morphology is still unclear. Testosterone affects a wide array of tissues and organs during development. Through altering testosterone levels, our implicated SNPs may impact the cell populations involved in facial growth and development. In this indirect model, testosterone acts as a mediator. However, testosterone levels are extremely variable during life, with a peak during puberty and a gradual decline thereafter (Harman et al., 2001). Also, there are immense intra-individual variation in testosterone levels, based on immune activation, caloric intake, interaction with other hormones, and several psychosocial variables (Harman et al., 2001). Therefore, the influence of testosterone levels on craniofacial growth is likely to be extremely complex. The testosterone-related genetic variants considered here were selected from GWAS studies on adults, while the vast majority of facial growth occurs prior to adulthood (Enlow, 1996). Different sets of genetic variants may affect testosterone levels in adults and children. This could be one reason many of the SNPs we investigated did not show effects on facial morphology. Testing genetic variants associated with circulating testosterone levels in subadults, during the active craniofacial growth phase, may yield improved results. Another possibility is that we lacked power to detect facial effects for some of the variants we tested. Power to detect testosterone-mediated genetic associations with facial morphology, which depends on the allele frequency of the variant, the effect size of the SNP on testosterone, and the effect size of testosterone on the face, differ across the SNPs tested in this study. Therefore, failure to detect an association should not be interpreted to mean that there is no effect.

Interestingly, some variants, in addition to impacting free testosterone levels, may also have a direct effect on facial morphology. Supporting this idea is a recent experimental study implicating *NR2F* genes in craniofacial morphogenesis, and, in particular, jaw development (Barske et al., 2018). However, there is currently no evidence supporting a similar role for *SHBG* or showing expression of this gene in developing facial tissues. Additional experimental follow-up studies will be required to understand the mechanisms through which these variants influence facial traits.

Several GWAS of normal-range facial morphology have now been published (Liu et al., 2012; Paternoster et al., 2012; Adhikari et al., 2016; Shaffer et al., 2016; Claes et al., 2018). Because these studies utilize an unbiased approach taking into account variants spread across the entire genome, they provide rich datasets to explore the influence of specific genetic pathways (in this case the testosterone pathway) on craniofacial morphology in a hypothesis-driven manner. Accordingly, the results here provide additional insights into the genetic basis of human facial features that would likely be missed in a typical GWAS, due to the very stringent adjustments for statistical significance required to control type-1 error inflation. This more targeted approach can help us identify biologically relevant genetic variants with more subtle effects on craniofacial morphology. That being said, because we identified our candidate SNPs from the literature, we are likely only testing a small fraction of the variants associated with testosterone levels (the ones that these studies had the power to detect) and we do not know the functional status of most of these variants. All of the variants associated with facial morphology in our study were non-coding. In future studies, it may be fruitful to consider interactions between variants implicated in this study and additional variants in other pathways reported to influence the same facial traits.

## Conclusion

We reported three SNPs associated to testosterone levels in the body with a clear effect on mandible shape (rs12150660 and rs1799941) and facial width to height ratios (rs8023580), indicating that testosterone-related genetic variants affect normal-range facial morphology, and in particular, facial features known to exhibit strong sexual dimorphism in humans.

## Data Availability

The datasets analyzed for this study are available through various sources. For the Pittsburgh dataset, the genotypic markers are available through the dbGaP controlled-access repository (http://www.ncbi.nlm.nih.gov/gap) at accession phs000949.v1.p1. The 3D facial images are available through the FaceBase Consortium (https://www.facebase.org/data/record/#1/isa:dataset/RID=14283). The participants making up the Penn State University dataset were not collected with broad data sharing consent. Given the highly identifiable nature of both facial and genomic information and unresolved issues regarding risk to participants, we opted for a more conservative approach to participant recruitment. Broad data sharing of these collections would thus be in legal and ethical violation of the informed consent obtained from the participants. This restriction is not because of any personal or commercial interests. Additional details can be requested from MS. The ALSPAC data will be made available to bona fide researchers on application to the ASLPAC Executive Committee.

## Ethics Statement

Institutional Review Board (IRB) approval was obtained at each recruitment site, and all participants gave their written informed consent before participation; for children, written consent was obtained from a parent or legal guardian. For the Pittsburgh sample, the following local ethics approvals were obtained: University of Pittsburgh IRB PRO09060553 and RB0405013; UT Health Committee for the Protection of Human Subjects HSC-DB-09-0508; Seattle Children’s IRB 12107; University of Iowa Human Subjects Office/IRB 200912764 and 200710721. For the Penn State sample, the following local ethics approvals were obtained: State College, PA (IRB 44929 and 4320); New York, NY (IRB 45727); Urbana-Champaign, IL (IRB 13103); Dublin, Ireland; Rome, Italy; Warsaw, Poland; and Porto, Portugal (IRB 32341); Twinsburg, OH (IRB 2503). For the ALSPAC sample, ethical approval for the study was obtained from the ALSPAC Ethics and Law Committee and the Local Research Ethics Committees.

## Author Contributions

KI, HH, and PC performed the modular analyses. ML performed the ratio analysis. JR wrote the first draft of the manuscript under supervision of SW, MM, EF, and JS. PC, JR, EF, JS, SW, and MM conceptualized the design of the study, with valuable feedback of CH-S. JR, EF, JS, SW, and MM organized the PITT cohort, with sample collection supported by JH, GW, and LM. JW and MS organized the PSU cohort and imputed the PSU genetic data. SR coordinated the collection of the ALSPAC images. JR, HH, MS, and JW provided input throughout the analyses and the writing process. All authors contributed to manuscript revision, read and approved the submitted version.

## Conflict of Interest Statement

The authors declare that the research was conducted in the absence of any commercial or financial relationships that could be construed as a potential conflict of interest.

## Footnotes

1 http://www.bris.ac.uk/alspac/researchers/data-access/data-dictionary/

2 http://www.ncbi.nlm.nih.gov/gap

## Supplementary Material

FIGURE S1 Five facial ratios tested for association with the candidate SNPs. **(A)** Total facial width to height ratio (Total FWH). **(B)** Upper facial width to height ratio – version 1 (Upper FWH1). **(C)** Upper facial width to height ratio – version 2 (Upper FWH2). **(D)** Lower facial width to height ratio (Lower FWH). **(E)** Upper to lower facial width ratio (Upper:Lower FW).

FIGURE S2 Results of the meta-analysis in the post-pubertal subset. Representation of the significant modules of the meta-analysis based on the different discovery datasets.

TABLE S1 List of 44 candidate SNPs selected from the literature.

TABLE S2 Complete meta-analysis results for all 32 SNPs tested on the 63 3D facial modules.

TABLE S3 Results for the 42 candidate SNPs tested for association with five facial ratios in the 3DFN cohort.
