# Supplementary figures and images for: SNPs Associated With Testosterone Levels Influence Human Facial Morphology
Source: Front Genet. 2018 Oct 23;9:497. doi: 10.3389/fgene.2018.00497 (PMC6206510; doi:10.3389/fgene.2018.00497)

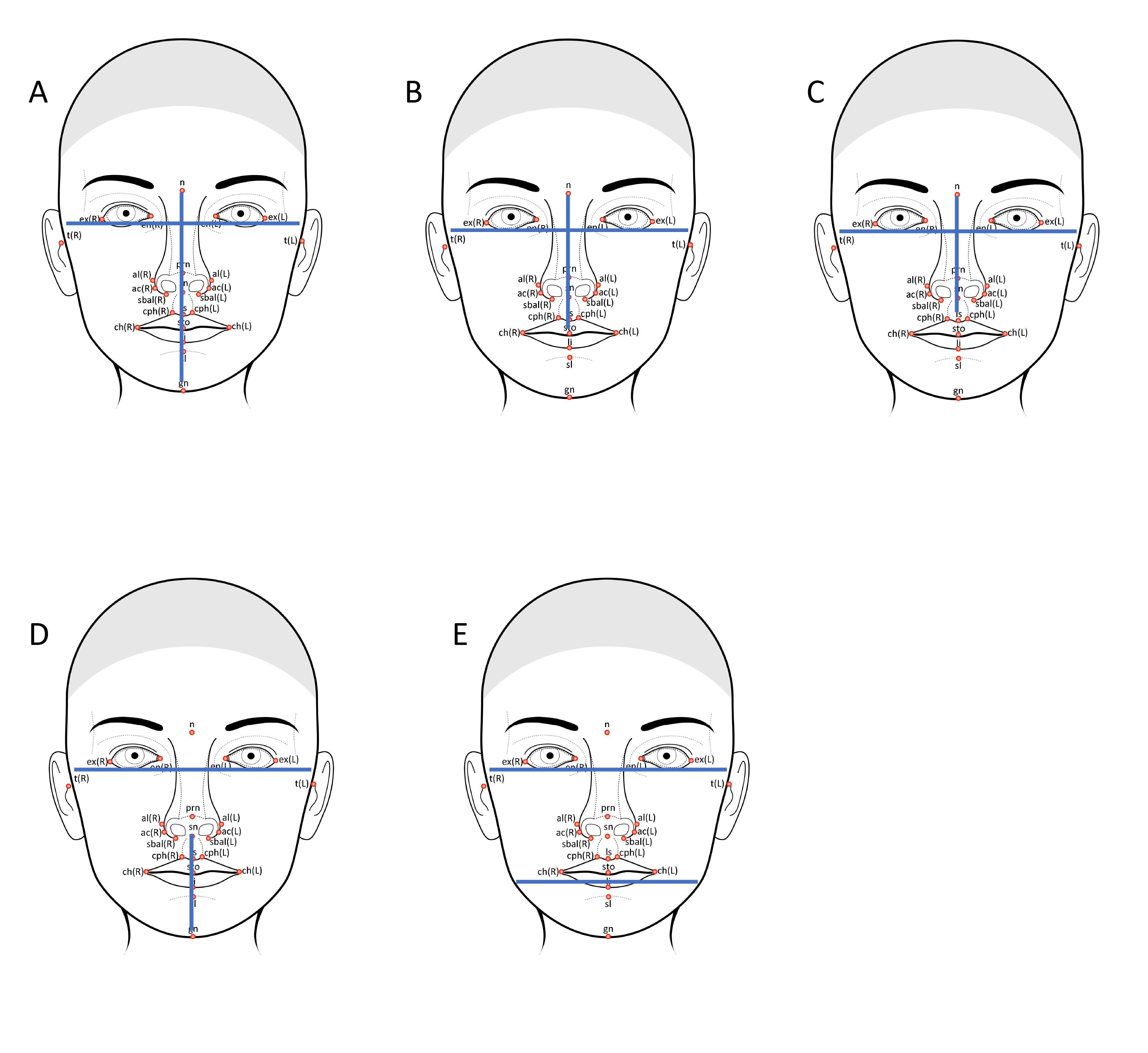

Supplement: FIGURE S1 — Five facial ratios tested for association with the candidate SNPs. (A) Total facial width to height ratio (Total FWH). (B) Upper facial width to height ratio – version 1 (Upper FWH1). (C) Upper facial width to height ratio – version 2 (Upper FWH2). (D) Lower facial width to height ratio (Lower FWH). (E) Upper to lower facial width ratio (Upper:Lower FW). [file Image_1.TIFF]

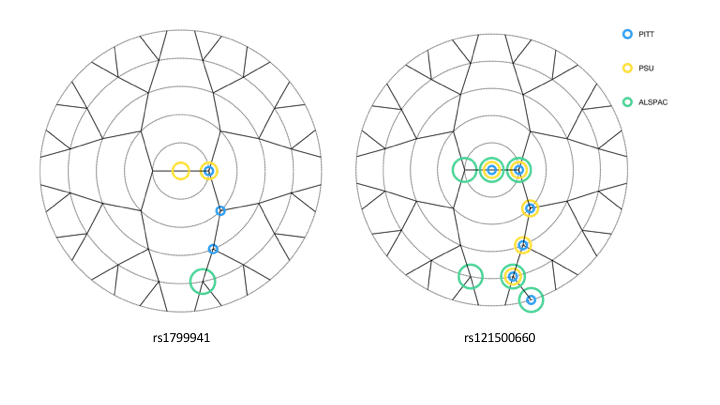

Supplement: FIGURE S2 — Results of the meta-analysis in the post-pubertal subset. Representation of the significant modules of the meta-analysis based on the different discovery datasets. [file Image_2.TIFF]
